# Dynamic contrast enhanced MRI of pulmonary adenocarcinomas for early risk stratification: higher contrast uptake associated with response and better prognosis

**DOI:** 10.1186/s12880-022-00943-x

**Published:** 2022-12-05

**Authors:** Stephan Rheinheimer, Petros Christopoulos, Stella Erdmann, Julia Saupe, Heiko Golpon, Jens Vogel-Claussen, Julien Dinkel, Michael Thomas, Claus Peter Heussel, Hans-Ulrich Kauczor, Gudula Heussel

**Affiliations:** 1 Diagnostic and Interventional Radiology with Nuclear Medicine, Thoraxklinik at University of Heidelberg, Röntgenstrasse 1, 69126 Heidelberg, Germany; 2 Diagnostic and Interventional Radiology, University Hospital Heidelberg, Im Neuenheimer Feld 420, 69120 Heidelberg, Germany; 3 Translational Lung Research Center Heidelberg (TLRC), University of Heidelberg, Im Neuenheimer Feld 420, 69120 Heidelberg, Germany; 4 Thoracic Oncology, Thoraxklinik at University of Heidelberg, Röntgenstrasse 1, 69126 Heidelberg, Germany; 5 Medical Biometry, Institute of Medical Biometry, Im Neuenheimer Feld 130.3, 69120 Heidelberg, Germany; 6 Department of Respiratory Medicine, Hannover Medical School, Carl-Neuberg-Str. 1, 30625 Hannover, Germany; 7 Diagnostic and Interventional Radiology and Biomedical Research in Endstage and Obstructive Lung Disease Hannover (BREATH), Carl-Neuberg-Str. 1, 30625 Hannover, Germany; 8 Institute of Diagnostic and Interventional Radiology, Hannover Medical School, Carl-Neuberg-Str. 1, 30625 Hannover, Germany; 9 Radiology, Asklepios Hospital Munich, Robert-Koch-Allee 2, 82131 Gauting, Germany; 10 German Center for Lung Research (DZL), Giessen, Germany

**Keywords:** Non-small-cell lung carcinoma, Early response, Treatment outcome, Response evaluation criteria in solid tumors, Magnetic resonance imaging, Perfusion, Protein-tyrosine kinases, Platinum, Survival analysis, Progression-free survival

## Abstract

**Background:**

To explore the prognostic value of serial dynamic contrast-enhanced (DCE) MRI in patients with advanced pulmonary adenocarcinoma undergoing first-line therapy with either tyrosine-kinase inhibitors (TKI) or platinum-based chemotherapy (PBC).

**Methods:**

Patients underwent baseline (day 0, n = 98), and post-therapeutic DCE MRI (PBC: day + 1, n = 52); TKI: day + 7, n = 46) at 1.5T. Perfusion curves were acquired at 10, 40, and 70 s after contrast application and analysed semiquantitatively. Treatment response was evaluated at 6 weeks by CT (RECIST 1.1); progression-free survival (PFS) and overall survival  were analysed with respect to clinical and perfusion parameters. Relative uptake was defined as signal difference between contrast and non-contrast images, divided by the non-contrast signal. Predictors of survival were selected using Cox regression analysis. Median follow-up was 825 days.

**Results:**

In pre-therapeutic and early post-therapeutic MRI, treatment responders (n = 27) showed significantly higher relative contrast uptake within the tumor at 70 s after application as compared to non-responders (n = 71, p ≤ 0.02), response defined as PR by RECIST 1.1 at 6 weeks. There was no significant change of perfusion at early MRI after treatment. In multivariate regression analysis of selected parameters, the strongest association with PFS were relative uptake at 40 s in the early post-treatment MRI and pre-treatment clinical data (presence of liver metastases, ECOG performance status).

**Conclusion:**

Higher contrast uptake within the tumor at pre-treatment and early post-treatment MRI was associated with treatment response and better prognosis. DCE MRI of pulmonary adenocarcinoma may provide important prognostic information.

**Supplementary Information:**

The online version contains supplementary material available at 10.1186/s12880-022-00943-x.

## Background

Risk stratification and early therapy response assessment are of key importance for patients with cancer, in order to guide subsequent management and avoid unnecessary toxicity and costs. Median survival of patients with advanced non-small-cell lung cancer (NSCLC) ranges from 1.5 to several years depending on mutation status [1]. The balance between treatment risk and therapeutic benefit is difficult to define in routine clinical practice. There are multiple factors to consider: comorbidities, patient preference, biology, and extent of metastatic spread. Of special interest in this regard are the so-called imaging biomarkers, which could predict tumor aggressiveness more precisely than routine staging procedures alone, while also avoiding the procedural risk associated with repeat biopsies and histopathologic evaluation. [2, 3]

Importantly, treatment response in targeted therapies may not be reflected appropriately by RECIST because of a different mechanism of action compared to direct cytotoxic agents [4, 5]. Therefore, morphological and functional imaging criteria have been explored for improved and earlier prediction of treatment response, such as volume reduction, change of tumor parameters including echogenicity, apparent diffusion coefficient, tissue perfusion, PET tracer accumulation, markers of ischemia [4, 6–13]. However, only few of these have been implemented in clinical decision-making algorithms thus far. For example, FDG uptake quantification is used for response evaluation in lymphoma [14], quantitative ultrasound parameters were found suitable for response assessment in breast cancer [15], and rectal cancer treatment response is evaluated by diffusion weighted imaging [16]. However, heterogeneity of tumor biology, small study cohorts and lack of standardization hampers validation of these criteria. Alongside PET/CT and perfusion CT, multiparametric MRI has shown promising initial results in characterization of pulmonary tumors [17] and assessment of treatment response [8, 18–21].

Contrast uptake is a widely accepted biomarker for tissue vitality and influenced by both tissue damage and vascular changes induced by the treatment [22, 23]. It is thought to correlate with tissue metabolism [4, 20]. Reduction in tumor perfusion has been shown in breast cancer under bevacizumab [24]. Similar effects have been described for different tumor entities under tyrosine-kinase inhibitors (TKI), like glioblastomas and colorectal cancer. Notably, these effects have been shown as early as two days after treatment initiation [24].

The present study investigates the prognostic information of serial dynamic contrast-enhanced magnetic resonance imaging (DCE MRI) in two histologically relatively homogeneous groups of patients with advanced pulmonary adenocarcinoma. Baseline and very early post-treatment contrast uptake curves under either platinum-based chemotherapy (PBC) or TKI were analyzed in conjunction with the subsequent clinical course.

## Materials and methods

This study was approved by the ethics committee of the medical faculty of Heidelberg (S-445/2015), and all participants provided written informed consent.

### Patients

**Fig. 1:**
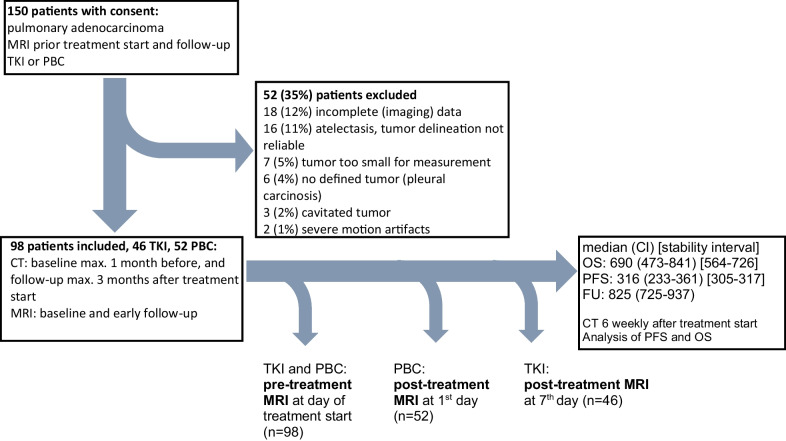
Flowchart of study patients

Between November 2016 and July 2019, 150 patients with advanced pulmonary adenocarcinoma and a measureable lesion of at least 2 cm in size under first line therapy were included in this prospective study (Fig. 1). Treatment was performed according to guidelines after consultation of the interdisciplinary tumor board. Patients undergoing radiation therapy of the primary tumor or local lymph nodes within the first 3 months were excluded. All included patients underwent pre-treatment and post-treatment MRI scans of high quality with few motion/pulsation artifacts, subjectively sufficient contrast enhancement and complete coverage of the primary tumor.

### Clinical documentation

Baseline patient and tumor characteristics were collected systematically from the medical records: body-mass-index (BMI), pulmonary function parameters, Eastern Cooperative Oncology Group (ECOG), smoking state including pack years and tumor biology (histology, mutation status, programmed death-ligand 1 (PD-L1) tumor proportion score; blood levels of the tumor markers carcinoembryonic antigen (CEA), Cytokeratin-fragment (Cyfra) 21.1, neuron-specific enolase (NSE), tumor stage (TNM 8th edition)).

All patients underwent routine CT and clinical work-up at maximum 4 weeks before and every 6 weeks after treatment initiation. RECIST 1.1 based response assessment was used as the gold standard [25]. Progression-free survival (PFS) was calculated as days between first MRI and follow-up CT with first progression or clinical progression in medical records. The imaging independent overall survival (OS) was calculated as days between first MRI and date of death.

### MR examination

According to our study design (Fig. 1), all MRI examinations of the lung were performed on the same 1.5T scanner (Magnetom Aera, Siemens, Erlangen, Germany). First MRI was performed at the day of treatment initiation (TKI orally daily or PBC intravenously every 3 weeks). Second MRI was performed one day after treatment start (PBC) or 1 week after treatment start (TKI).

**Fig. 2:**
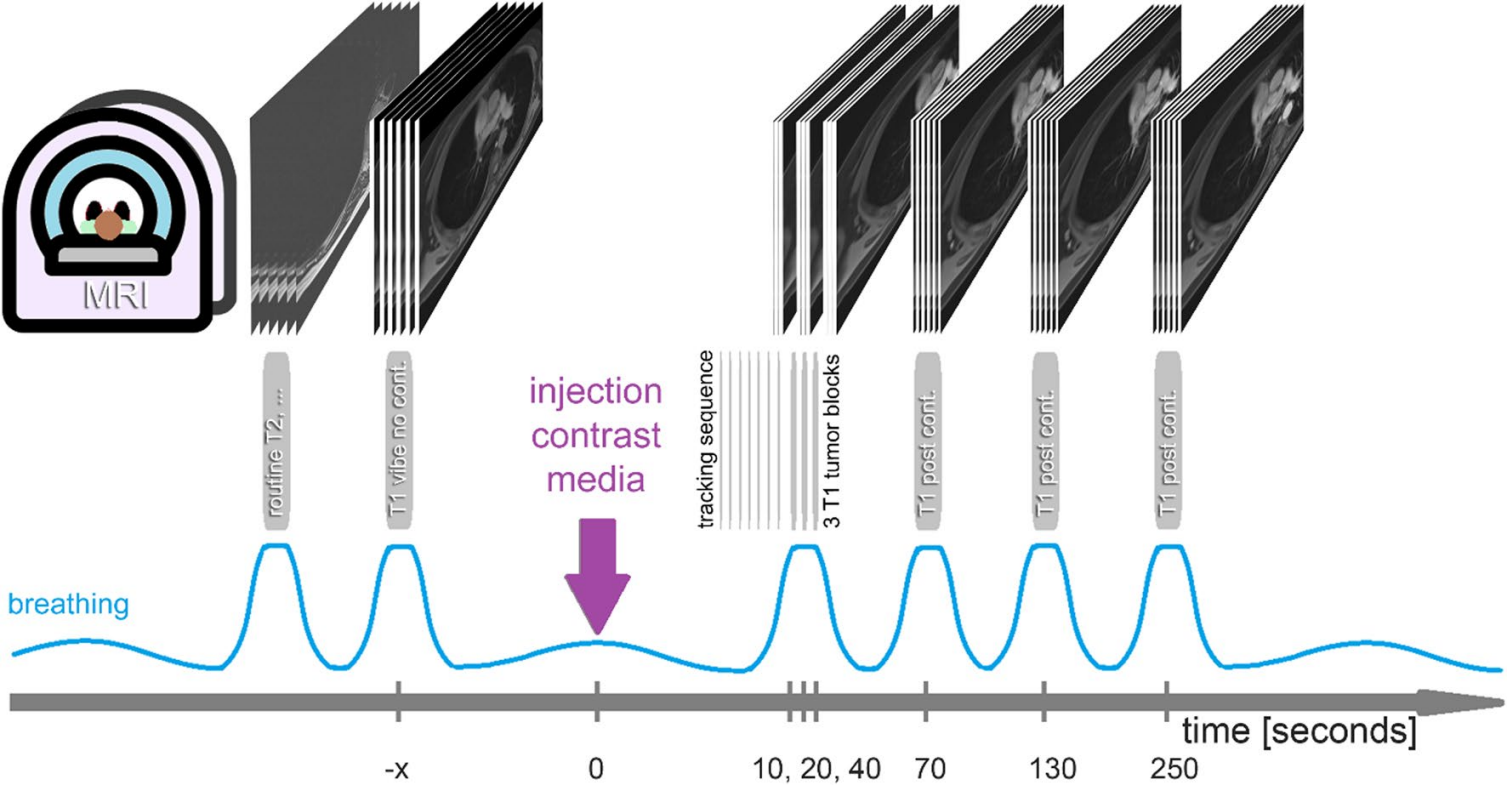
MRI protocol: note, that at time point 10 s, 20 s, and 40 s only a small image stack was obtained covering the tumor, whereas all other time points are covering the whole thorax

Axial 3D volumetric interpolated breath-hold gradient echo T1 weighed fat saturated (frequency selective) dynamic contrast-enhanced sequences (T1 vibe) were acquired with the following parameters: 24 slices of matrix 320 × 180 pixels, slice thickness 4 mm, pixel bandwidth 540 Hz, repetition time 3.6 s, echo time 1.65s, flip angle 5°. This resulted in an acquisition time of 10 s for 24 slices and 30 s for 80 slices. After non-contrast series, contrast media was injected via a cubital vein with a flow of 1.5 ml/s followed by a 30 ml chaser bolus (1 mmol/kg body weight gadobutrol; Bayer, Leverkusen, Germany). Dynamic imaging sequences were triggered by bolus tracking sequence in the pulmonary trunk in coronal plane. In one single 30 s long breath hold, three repeated small image stacks covering the primary tumor with 24 images were obtained 10 s, 20 and 40 s after contrast administration (Fig. 2). At 70 s, 130 and 250 s delay whole thorax imaging (80 images each) was performed, each during separate 30 s breath holds. Note that time between contrast administration is simplified as a uniform 10 s interval. Time steps are 0 s (non-contrast), 10 s, 20 s, 40 s, 70 s, 130 and 250 s. Breath holding was instructed automatically between the sequences [26]. Overall MR acquisition time was around 15 min.

### CT examination

CT scans (max. 3 mm slices, no motion artifacts, at maximum 1 month before treatment start) were obtained as part of routine clinical care. Most scans were obtained with a Somatom Definition AS64 scanner (Siemens, Erlangen, Germany) with application of iodinated contrast media.

### Image analysis

**Fig. 3:**
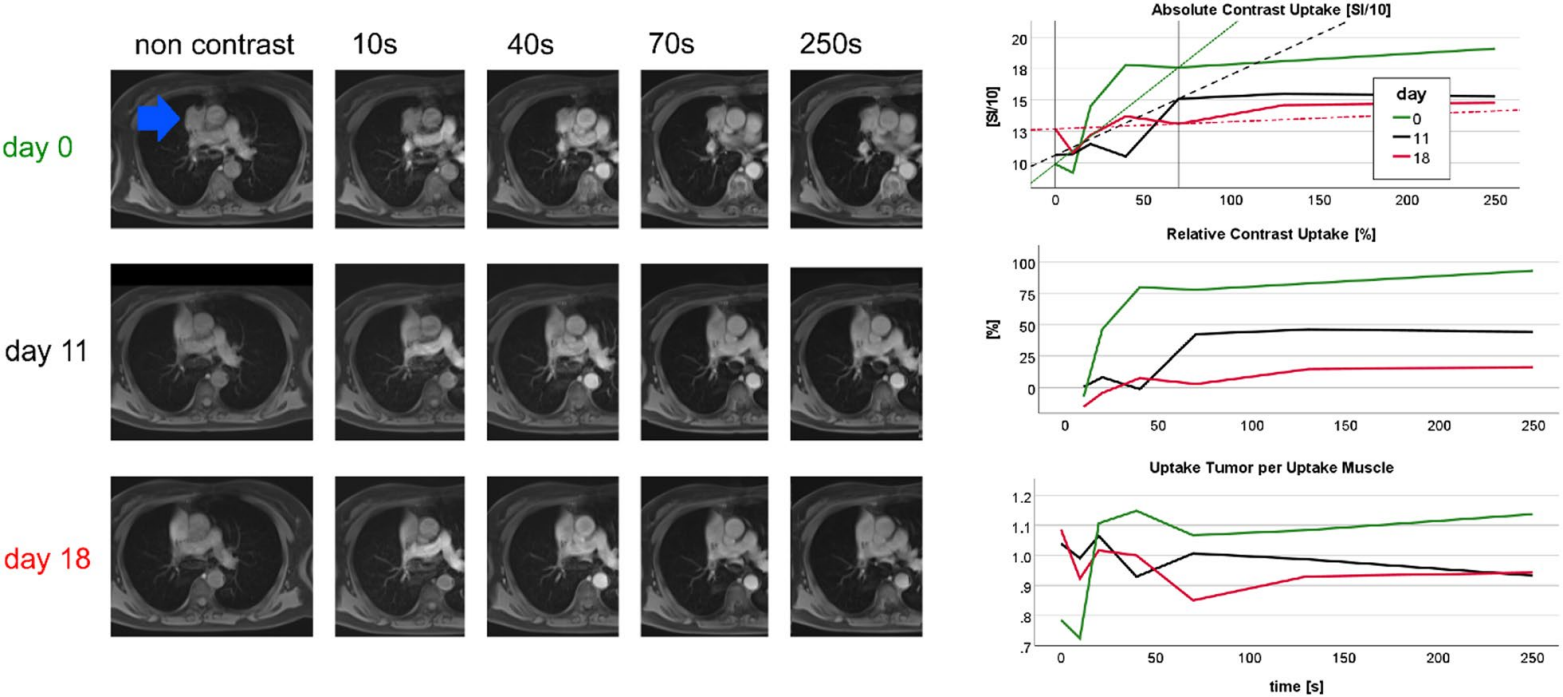
DCE MRI of a 69 year-old female non-responder, smoker (15 pack years), adenocarcinoma right upper lobe (blue arrow), T3N1M1(Oss), received TKI, progression-free survival 117 days (progression by new lymph node metastases), overall survival 143 days. Pre-therapeutic strong uptake followed by post-therapeutic reduced uptake accompanied by early progression and short survival. *Note*: MR2 at day 11 due to scheduling delay, MR3 given additionally. Left: representative time points of DCE MRI pre-therapeutic (day 0) and post-therapeutic. Right: semiquantitative contrast enhancement curves (above absolute SI of tumor, middle relative contrast enhancement, below contrast normalized tumor to muscle SI).

**Fig. 4:**
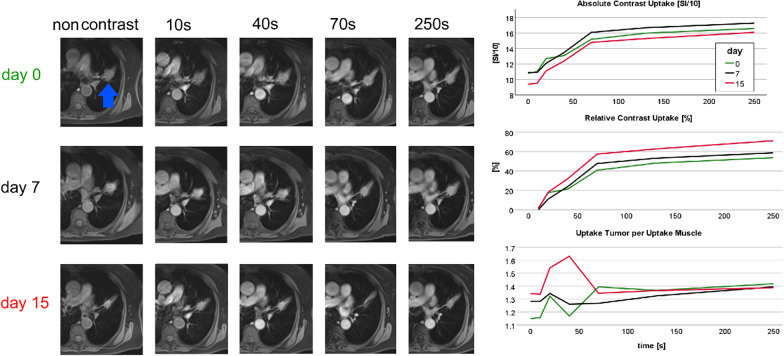
DCE MRI of a 81 year-old female responder, never smoker, adeno carcinoma left upper lobe (blue arrow), T4N1M1(Hep, Oss), received TKI, progression-free survival 234 days (progression with new liver metastases), overall survival 1182 days. This relatively long PFS/OS goes along with minimal increase of contrast enhancement. This is in line with calculated negative association of relative uptake after 40 s in post-therapeutic MRI and PFS.

**Fig. 5:**
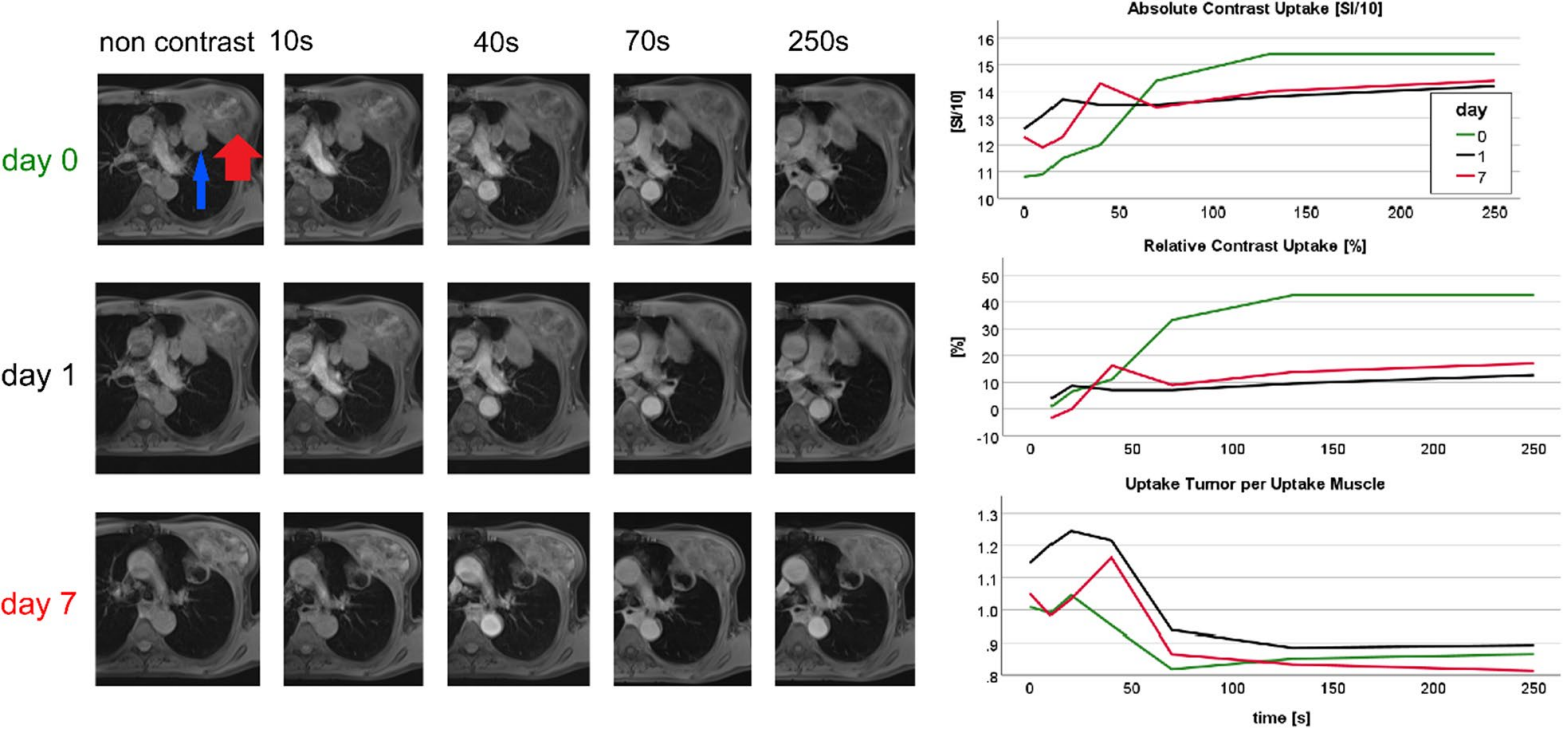
72 Year-old male responder, smoker (50 pack years), adeno carcinoma left upper lobe (blue arrow), T3N2M1(Adr, Oss), received PBC, lost in follow-up after 79 days without progression. At day 7 central necrosis in the tumor is seen. Initially, the tumor shows moderate contrast uptake. This is reduced early after therapy and necrosis is visible at 70 s post contrast injection and later. Note the huge costal metastasis, which changed minimally during the course of therapy (red arrow)

To compensate for respiration-related misplacement for each time step of DCE-MRI, a free-hand region of interest (ROI) was placed around the whole tumor at the level of widest tumor diameter, sparing airways and vessels. Care was taken in each examination pre- and post-treatment that the ROI was placed in an equivalent anatomical position. ROI area was recorded for each MRI. As reference, ROIs were placed in pectoral muscle, normalized enhancement curves exemplary shown in Figs. 3, 4 and 5. MR analysis for pre-treatment and post-treatment measurement and documentation took around 30 min. ROI placement was performed in our routine image viewer (Synapse© PACS, Fujifilm, Minato, Japan) results were documented in Microsoft Excel® 2019 (Redmond, Washington, USA). Internal reproducibility was confirmed by a single observer. In 16 patients repeated measurements were carried in a time interval of 6 months. Interclass correlation coefficient was between 0.96 and 0.99 for signal ratios at 0 s, 40 s, 70 s, relative uptake at 40 s and at 70 s, and for the slope values (explained in the next section).

The following semiquantitative parameters were calculated from perfusion curves: relative contrast uptake at 40 and 70 s, maximal uptake, wash-in contrast kinetic (0 to 40 s, 0 to 70 s). Relative tumor uptake (Rel. UT) was calculated according to the following formula: Rel. UT = (SI_t_ – SI_0_)/SI_0_, where SI_t_ is tumor signal intensity at time t and SI_0_ is tumor signal intensity before contrast administration. As surrogate for total contrast enhancement, the area under the curve (AUC) was calculated as the sum of the mean signal for each time interval multiplied by that time interval over the range of 0–250 s. Image processing and documentation of clinical data and imaging were done by expert thoracic radiologists (at least 8 years of experience) and thoracic oncologists (at least 15 years of experience).

### Statistics

Baseline variables are descriptively compared for both groups (responders, non-responders). Depending on the variable, mean ± standard deviation or absolute and relative frequencies are given. Associated p-values are calculated by Student’s t-test, Welch’s t-test, or Chi-Square test, respectively. We report the median follow-up time calculated by the inverse Kaplan–Meier method with corresponding 95% confidence intervals and “stability interval” as suggested by Schemper and Betensyk, respectively [27, 28].

In order to assess the potential additional benefit of imaging parameters, a combination of forward and backward selection procedure (the FAMoS Algorithm) based on the AIC (Akaike information criterion) was used for model selection [29]. To construct a robust multivariate model for our study group of 98 patients, we performed the model selection in three steps: First, we performed a variable selection on a data set containing complete observations on all relevant clinical variables (therapy group, age, gender, abnormal body mass index, clinical status, smoking status, Cyfra 21.1, EGFR status, tumor stage and presence of liver metastases). The variables selected in this step were included in the starting model. In the second step pre-therapeutic MRI variables could be included (forward selected), but clinical parameters could be excluded (backward selection), based on a data set containing all information on the relevant variables. In the third step, again the selected variables from the step before were included in the starting model. Post-therapeutic MRI variables were included if relevant and previously selected variables could be excluded based on the AIC criterion and a data set which contained all information on the relevant variables. The model was applied to OS and PFS respectively, and the group variable (TKI, PBC) was always included in the model. The resulting Cox regression models are presented by means of the hazard ratios (HR) and associated 95% confidence intervals and descriptive p-values of the selected variables, as well as the AIC, number of observations and events in the model.

A p-value of < 0.05 was considered as statistically significant. Missing values were not imputed, resulting in complete case analysis with respect to the specific analysis. Analysis was done using R Version 4.0.2 (30) and SPSS Version 27, IBM, Armonk, USA. In order to facilitate better understanding of the calculated hazard ratios, slope values were multiplied by 10 to report a clinically relevant scale.

## Results

98 patients with sufficient imaging and clinical data were finally included into the study, 46 patients TKI group (15 male) and 52 patients with PBC (27 male). At 6 weeks, 27 (4 PBC, 23 TKI) showed partial treatment response. Responders and non-responders had generally similar baseline characteristics, with one notable exception: more never smokers responded (Table 1). All six patients without metastases (stage III disease) showed no response after 6 weeks of treatment.

**Table 1:** Patient characteristics

		Responders* (n = 27)	Non-responders* (n = 71)	Total population (n = 98)
TKI/PBC		23/4^¤^	23/48^¤^	46/52
Post-treatment MRI [days]		8.6 ± 4.4^¤^	3.9 ± 4.3^¤^	5.2 ± 4.8
Mean age		64 ± 9	64 ± 9	64 ± 9
Male		10 (37%)	32 (45%)	42 (43%)
ECOG > 0		15 (56%)	28 (39%)	43 (44%)
Pathologic BMI^♦^		4 (15%)	19 (27%)	23 (24%)
Never Smoker		10 (37%)^×^	10 (14%)^×^	20 (20%)
Pack Years		15 ± 18^¤^	33 ± 23^¤^	28 ± 23
Vital capacity [l]		2.8 ± 1.1	3.0 ± 1.0	3.0 ± 1.0
Baseline CEA [ng/ml]		294 ± 1259	97 ± 241	149 ± 675
Baseline Cyfra 21.1 [ng/ml]		9.1 ± 11.3	9.1 ± 10.5	9.1 ± 10.7
Baseline NSE [ng/ml]		35 ± 24	27 ± 27	29 ± 27
*Tumor*				
Stage III		0 (0%)	6 (8%)	6 (6%)
Stage IV		27 (100%)	65 (92%)	92 (94%)
*T-stage*				
T1		2 (7%)	7 (10%)	9 (9%)
T2		5 (19%)	18 (25%)	23 (24%)
T3		5 (19%)	18 (25%)	23 (24%)
T4		14 (52%)	28 (39%)	42 (43%)
*N-stage*				
N1 and N2		16 (59%)	42 (59%)	58 (59%)
N3		11 (41%)	29 (41%)	40 (41%)
*M-stage*				
M0		0 (0%)^×^	6 (8%)^×^	6 (6%)
1 site		4 (15%)	23 (32%)	27 (28%)
2 sites		11 (41%)	21 (30%)	32 (33%)
≥ 3 sites		12 (44%)	21 (30%)	33 (34%)
*Metastases*				
Liver		5 (19%)	16 (23%)	21 (21%)
Brain		9 (33%)	24 (34%)	33 (34%)
Bone		17 (63%)	31 (44%)	48 (49%)
Lung		13 (48%)	22 (31%)	35 (36%)

Categorial variables in absolute values (relative value) tested by Chi Square test, continuous variables in means ± SD tested by Welch’s t-test

*Defined as RECIST 1.1 PR in first follow-up CT

^+^Defined as RECIST 1.1 SD or PD in first follow-up CT

^♦^BMI < 20 or > 30; ^×^P = 0.005; ^¤^P < 0.001

In pre-treatment MRI, lung tumors of responders presented a significantly higher contrast uptake 70 s after contrast administration compared to non-responders (Table 2). Consequently, the slope of contrast curve was also higher. In the early post-treatment MRI, differences of contrast uptake were more pronounced: other additional parameters, such as relative contrast uptake 40 s after administration, slope at 40 s, maximum contrast uptake, and AUC were significantly higher in responders. Except for ΔAUC, pre-treatment to post-treatment differences of these parameters were not significant, indicating no measurable treatment effect on the present contrast curves. Notably, in responders, there was a significant reduction of ROI area between pre- and post-treatment MRI after 5.2 ± 4.8 (range 1 to 18) days. Patients that received TKI presented tumors with higher perfusion values compared to patients witch received PBC.

**Table 2:** Comparison responder (RECIST 1.1 PR at 6 week CT) and non-responder (SD or PD at 6 week CT)

	Responders* (n = 27)	Non-responders^+^ (n = 71)	P-value^×^
*General features*			
Sum of diameter CT [cm]	7.7 ± 4.6	8.4 ± 3.9	0.44
Mean PFS ± SD [days]	401 ± 211	317 ± 230	0.10
Mean OS ± SD [days]	706 ± 320	508 ± 293	**0.004**
*Pre-therapeutic MRI*			
40 s rel. uptake [%]	33.7 ± 15.6	28.4 ± 17.9	0.18
Slope 0–40 s [*10]	9.4 ± 4.7	7.9 ± 4.9	0.19
70 s rel. uptake [%]	49.0 ± 17.9	35.9 ± 25.6	**0.02**
Slope 0–70 s [*10]	7.7 ± 2.4	5.6 ± 3.6	**0.006**
Max. uptake [SI]	171 ± 24	162 ± 32	0.22
AUC [SI/250 s]	4029 ± 551	3849 ± 731	0.24
*Post-therapeutic MRI*			
40 s rel. uptake [%]	33.4 ± 12.0	25.3 ± 17.1	**0.03**
Slope 0–40 s [*10]	9.7 ± 3.7	7.1 ± 4.6	**0.01**
70 s rel. uptake [%]	47.3 ± 22.4	32.4 ± 24.1	**0.007**
Slope 0–70 s [*10]	7.7 ± 3.2	5.0 ± 3.3	**< 0.001**
Max. uptake [SI]	182 ± 22	162 ± 25	**< 0.001**
AUC [SI/250 s]	4264 ± 509	3814 ± 610	**0.001**
*Difference MRI pre-MRI post therapy*			
Area difference MR1-MR2 [cm^2^]	3.1 ± 4.1	0.6 ± 2.5	**< 0.001**
Δ 40 s rel. uptake [%]	0.3 ± 13.3	2.7 ± 15.8	0.49
Δ Slope 0–40 s [*10]	– 0.4 ± 3.4	0.7 ± 4.2	0.24
Δ 70 s rel. uptake [%]	2.7 ± 22.1	2.7 ± 21.1	0.99
Δ Slope 0–70 s [*10]	0.0 ± 2.8	0.5 ± 3.0	0.52
Δ Max. uptake [SI]	– 9 ± 22	0 ± 20	0.05
Δ AUC [SI/250 s]	– 184 ± 370	29 ± 474	**0.04**

Bold means* P*-value < 0.05 was considered to be significant

^×^Means ± SD tested by students t-test

*Defined as RECIST 1.1 PR in first follow-up CT

^+^Defined as RECIST 1.1 SD or PD in first follow-up CT

**Fig. 6:**
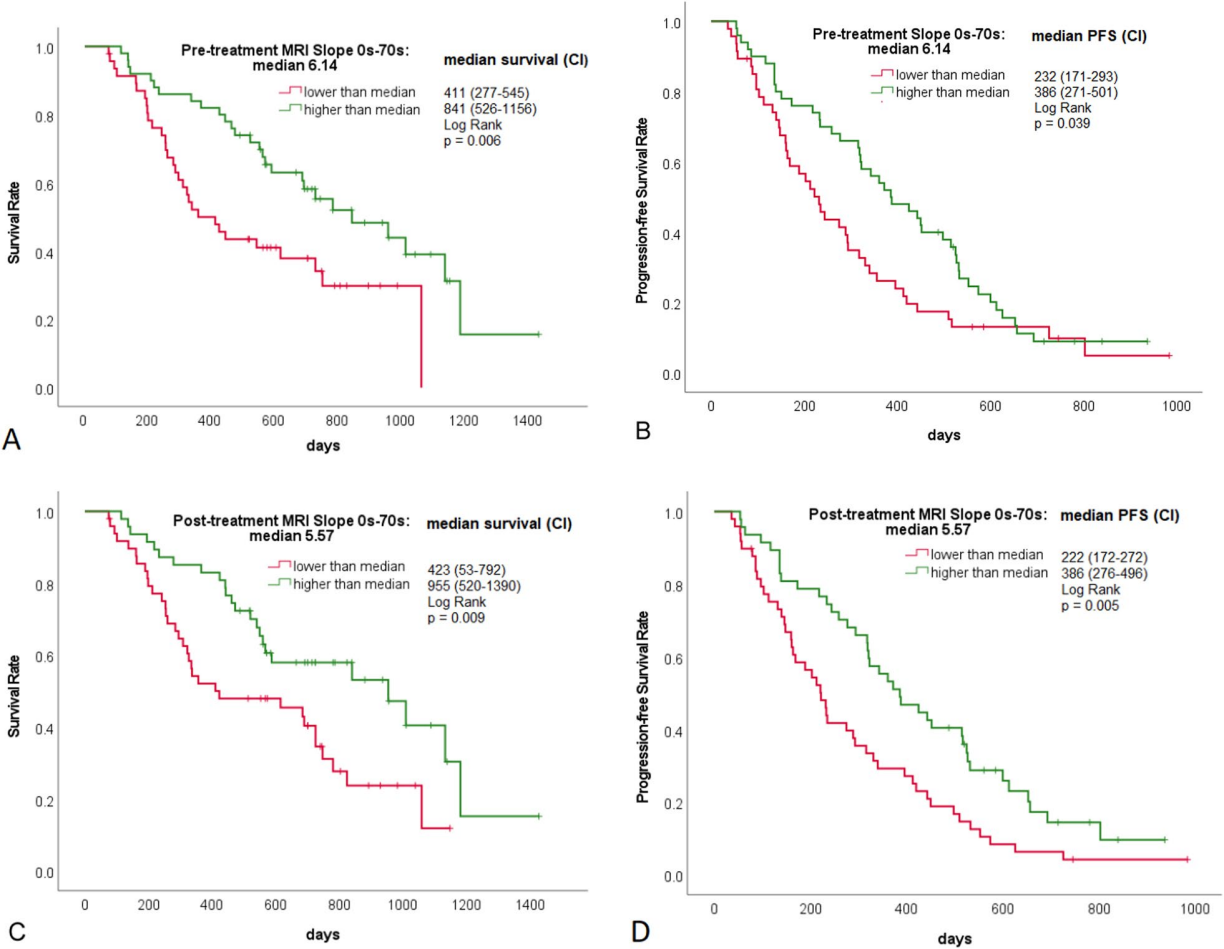
Kaplan–Meier plots: OS (**A**, **C**) and PFS (**B**, **D**) dependent on pre-treatment contrast uptake (**A**, **B**) and early post-treatment contrast uptake (**C**, **D**)

Figures 3, 4 and 5 illustrate three representative cases. The tumor of a TKI non-responder showed a 75% uptake at 70 s after contrast administration that dropped stepwise under treatment (Fig. 3). In contrast to this, a TKI responder showed an initial relatively low uptake of 40% at 70 s, discretely increasing to 60% (Fig. 4), while a responder to PBC treatment with central tumor necrosis presented a perfusion reduction (Fig. 5). Figure 6 demonstrates higher mortality (A, C) and shorter progression-free survival (B, D) of patients with contrast uptake below median.

### Univariate analyses of clinical factors, pre-therapeutic imaging and post-therapeutic imaging

The relationship between clinical, pre-therapeutic imaging and post-therapeutic imaging parameters with PFS and OS were analyzed using univariate Cox regression (Additional file 1: Table A.1). There was a significant association with several clinical parameters as well as pre-treatment and post-treatment imaging parameters.

### Model selection and multivariate analyses

Using forward and backward selection procedures, four clinical parameters with optimally combined PFS or OS prediction were selected (Additional file 1: Table A. 2 for OS and Additional file 1: Table A. 3 for PFS, first row). In the second step, best model fit was achieved using slope 0–70 s for OS. For PFS, pre-therapeutic MRI did not lead to a better model fit (Additional file 1: Table A. 2 for OS and Additional file 1: Table A. 3 for PFS, second row). In the third step, the post-therapeutic relative uptake value at 40 s lead to a better model fit for PFS (Additional file 1: Table A. 3). In contrast, for OS, results of the post-therapeutic MRI did not result in significant improvement of the model (Additional file 1: Table A. 2).

## Discussion

Our study uses semiquantitative contrast wash-in kinetic parameters for description of pre-therapeutic and very early post-therapeutic DCE MRI in 98 adenocarcinomas of the lung. To the best of our knowledge, this is the first study of purely advanced adenocarcinomas of the lung that evaluates early MRI perfusion changes under PBC or TKI therapy. A long follow-up interval allowed regression analysis not only to mainly imaging dependent parameters as RECIST and PFS but also to overall survival. Inclusion criteria were broad, and as such quite representative for a clinical real-life setting.

Main finding of our study is a significantly higher tumor perfusion of responders compared to non-responders in pre-therapeutic and early post-therapeutic MRI, which were clearly associated to PFS and OS and therefore predicts outcome before treatment start. This confirms former studies, which have also described the relationship between stronger baseline perfusion with better treatment response [8, 19, 31]. For example, Fraioli et al. demonstrated a higher baseline blood flow in 11 responders compared to 34 non-responders in 45 patients with advanced adenocarcinoma using CT perfusion [32]. Tissue perfusion may increase therapy susceptibility as capillarization is mandatory for exposure to therapeutic agents. Possibly, stronger perfused adenocarcinomas might also represent a less aggressive tumor biology as these malignancies may contain fewer microscopic necrotic areas. In our cohort, patients with positive EGFR status and TKI treatment showed higher perfusion values and a higher response rate. Although this is a confounding factor, our multivariate analyses demonstrate treatment independent association of baseline perfusion and prognosis.

We could not show clear treatment related changes of MRI parameters in this early phase of treatment, whereas the area reduction of the tumor was significantly higher in responders compared to non-responders. Therefore, in the setting of PBC or TKI without additional antiangiogenics, treatment-related changes were clinically informative only regarding size, but not functional parameters of the tumor. These results are similar to those of other studies, which have observed inferior predictive capacity for perfusion compared to metric changes of the tumor in several tumors, including lung and breast cancer [4, 33]. In contrast, in studies combining PBC with antiangiogenic treatment, blood flow as assessed by CT was reduced after one or more cycles of therapy in responders [32, 34, 35].

Several quantitative DCE MRI studies of small and heterogeneous cohorts have documented reduced perfusion in treatment responders [6, 8, 19]. This finding is explained by tumor tissue damage due to reduced angiogenesis. Contrary to this, treatment-associated inflammation could increase tissue perfusion in the early phase of therapy. Differences in timing might explain conflicting results of studies. As prognostic marker, Tao et al. evaluated deconvolution perfusion MRI before treatment in 36 NSCLC patients, of which 6 were adenocarcinomas [19]. Response was evaluated after completion of radiation therapy after 1 month. Responders showed higher baseline k_trans_ and lower baseline k_ep_ and V_e_. Chang et al. also identified prognostic impact of baseline perfusion markers in 11 NSCLC patients of whom 10 suffered from adenocarcinoma. In contrast to the data of Tao et al., high k_ep_ correlated with response. Similar to Tao et al., low V_e_ was predictive for response. As predictive parameter, k_trans_ reduction correlated with tumor diameter reduction after three cycles of chemotherapy [8]. Similarly, Xu et al. showed as early as 1 week after classic chemotherapy initiation a significantly reduced k_trans_ and V_e_ in 13 treatment responders compared to 9 non-responders [6]. This study included 11 patients with adenocarcinomas.

No predictive impact of change of k_trans_ was shown by de Langen et al. in 28 patients with non-squamous NSCLC 3 weeks after starting antiangiogenic therapy. In histogram analysis, increase of standard deviation of k_trans_ over 15% was associated with treatment failure [4]. Based on these studies, strong baseline tumor perfusion is a positive prognostic marker for NSCLC. Perfusion decrease under treatment seems to correlate with response, but study results differ in this point, potentially due to differences in tumor biology, treatment and timing of imaging. On the whole, OS as an end-point metric criteria other than RECIST have only be defined in a few NSCLC studies [4, 31]. Therefore, in most studies superiority of perfusion parameters to RECIST is not assessable and the benefit of this independent predictive marker additional to early RECIST assessment remains unclear.

To assess the interaction of different prognostic factors, multiparametric Cox regression was applied. In order to reduce the problem of multiple statistical testing, we performed a three-step variable pre-selection for multivariate analyses. Our multivariate variable selection model indicates a better OS prediction with parameters of pre-therapeutic and post-therapeutic MRI and a better PFS prediction with parameters of post-therapeutic MRI, additional to selected clinical parameters. Therefore, perfusion MRI of pulmonary adenocarcinomas may supplement peri-therapeutic risk stratification.

Some important limitations of our study must be acknowledged:

1. One third of the patients have been excluded, most of them due to incomplete data, inferior imaging quality (i.e. low contrast enhancement) or scheduling delay of examinations. Other patients were excluded due to limitations in making tumor measurements, namely tumor atelectasis, diffuse tumor manifestation or too small tumor size. We believe that this exclusion process lead to more robust data analysis, but some exclusion criteria are subjective and confounding effects cannot be excluded. Reduced sample size was not suitable for evaluation of treatment subgroups.
2. Our perfusion approach was a simplified method using breath hold technique without calculation of tissue permeability parameters addressing the limitations of patients with severe pulmonary diseases. The present method has low temporal resolution but high spatial coverage and high contrast resolution than other methods. Time interval of contrast administration to first image series was not documented and this interval was assumed to be 10 s. Therefore, this very early interval is confounded by individual circulation differences of the patients. Review of perfusion curves confirmed sufficient plot of contrast kinetics. For semiquantitative parameters, similar significance levels for perfusion changes in NSCLC were achieved compared to quantitative calculation [21]. Semiquantitative perfusion curve description is easy to perform and robust, whilst quantitative calculation may underlie high variation [21]. Criteria might easily be translated to different imaging techniques like CT and to different study centers. Future free-breathing sequences may provide higher temporal resolution. This may optimize data quality especially in the pre-contrast phase and the inflow phase and might help to calculate reliable tissue specific parameters.
3. Free-hand ROI placement was carried out in one single layer and no histogram analyses were performed. Therefore, tumor changes could be underestimated. Free-hand ROI placement was necessary to compensate for different respiratory positions of the tumor. Tested automatic and semi-automatic registration algorithms were not sufficient to compensate for these movements. Intraobserver reproducibility was excellent, whereas interoberserver reproducibility was not tested in this study.
4. Only primary tumors were measured. This may not represent the prognostic most relevant tumor location. This aspect is less relevant in the first line therapy setting. Primary tumors did not undergo local therapies and systemic therapy effects should be evaluable at this site.
5. Progression-free survival and overall survival are confounded by treatment changes in later course. However, treatment was not changed until first follow-up CT after 6 weeks. Only a minority of patients underwent treatment change before fulfilling criteria of RECIST progress due to individual treatment regimes.

## Conclusion

Better tumor perfusion of pulmonary adenocarcinomas predicts response before and also shortly after treatment start and is independently associated with better prognosis.

## Supplementary Information

**Additional file 1**. Supplementary tables.

## Data Availability

The raw data cannot be made freely available because of privacy restrictions but the datasets used and/or analyzed during the current study are available from the corresponding author on reasonable request.

## Abbreviations

AIC: Akaike information criterion
AUC: Area under the curve
CEA: Carcinoembryonic antigen
CI: Confidence interval
CT: Computed tomography
Cyfra: Cytokeratin-fragment
DCE MRI: Dynamic contrast-enhanced magnetic resonance imaging
ECOG: Eastern Co-operative Oncology Group
EGFR: Epidermal growth factor receptor
FAMoS: Forward and backward selection procedure
FU: Follow-up
HR: Hazard ratio
NSCLC: Non-small-cell lung cancer
NSE: Neuron-specific enolase
OS: Overall survival
PBC: Platinum based chemotherapy
PET: Positron emission tomography
PFS: Progression-free survival
RECIST: response evaluation criteria in solid tumors
Rel. UT: Relative uptake
TKI: Tyrosine-kinase inhibitor
