# Supplementary material for: Dynamic contrast enhanced MRI of pulmonary adenocarcinomas for early risk stratification: higher contrast uptake associated with response and better prognosis
Source: BMC Med Imaging. 2022 Dec 5;22:215. doi: 10.1186/s12880-022-00943-x (PMC9724354; doi:10.1186/s12880-022-00943-x)
Supplement: Supplementary file 1 — Additional file 1. Supplementary tables. [file 12880_2022_943_MOESM1_ESM.docx]

Supplementary tables

Supplementary Table A.1. Univariate Cox regression of overall survival and progression free survival total population

| Total Population |  |  | Overall Survival (59 events) | | Progression Free Survival (87 events) | |
| --- | --- | --- | --- | --- | --- | --- |
|  | n | Mean or rate | P-value | Hazard Ratio (95% CI) | P-value | Hazard Ratio (95% CI) |
| Therapy group: TKI | 98 | 46/98 | 0.358 | 0.781 (0.468-1.307) | 0.011 | 0.573 (0.373-0.881) |
| No liver metastases | 98 | 77/98 | 0.012 | 0.490 (0.282-0.853) | 0.002 | 0.449 (0.269-0.748) |
| ECOG >0 | 97 | 43/95 | 0.017 | 1.887 (1.119-3.180) | 0.019 | 1.694 (1.091-2.631) |
| Cyfra 21.1 [ng/ml] | 84 | 9.1 | <0.001 | 1.036 (1.016-1.056) | 0.001 | 1.029 (1.012-1.047) |
|  |  |  |  |  |  |  |
| Pre-therapeutic MRI |  |  |  |  |  |  |
| 40s post CM [SI/10] | 98 | 14.62 | 0.125 | 0.930 (0.848-1.020) | 0.031 | 0.921 (0.855-0.993) |
| 40s rel. uptake [%] | 98 | 29.9 | 0.088 | 0.986 (0.971-1.002) | 0.247 | 0.993 (0.981-1.005) |
| Slope 0s-40s [*10] | 98 | 8.32 | 0.058 | 0.948 (0.898-1.002) | 0.105 | 0.966 (0.927-1.007) |
| 70s post CM [SI/10] | 97 | 15.60 | 0.006 | 0.872 (0.791-0.962) | 0.004 | 0.893 (0.826-0.965) |
| 70s rel. uptake [%] | 97 | 39.6 | 0.011 | 0.984 (0.972-0.996) | 0.158 | 0.993 (0.984-1.003) |
| Slope 0s-70s [*10] | 97 | 6.15 | 0.003 | 0.879 (0.808-0.957) | 0.029 | 0.929 (0.869-0.993) |
|  |  |  |  |  |  |  |
| Post-therapeutic MRI |  |  |  |  |  |  |
| 40s post CM [SI/10] | 97 | 14.74 | 0.048 | 0.911 (0.830-0.999) | 0.015 | 0.902 (0.831-0.980) |
| 40s rel. uptake [%] | 97 | 27.6 | 0.017 | 0.986 (0.965-0.997) | 0.003 | 0.981 (0.968-0.994) |
| Slope 0s-40s [*10] | 97 | 7.85 | 0.008 | 0.929 (0.880-0.981) | 0.003 | 0.933 (0.892-0.977) |
| 70s post CM [SI/10] | 96 | 15.65 | 0.028 | 0.899 (0.817-0.989) | 0.007 | 0.898 (0.829-0.971) |
| 70s rel. uptake [%] | 96 | 36.4 | 0.108 | 0.991 (0.979-1.002) | 0.034 | 0.990 (0.981-0.999) |
| Slope 0s-70s [*10] | 96 | 5.75 | 0.045 | 0.925 (0.858-0.998) | 0.013 | 0.925 (0.869-0.984) |

Supplementary Table A.2. Multivariate Cox Regression of FAMoS selected variables referring to OS

|  | P-value | Hazard Ratio  (95% CI) | P-value | Hazard Ratio  (95% CI) | P-value | Hazard Ratio  (95% CI) |
| --- | --- | --- | --- | --- | --- | --- |
|  | Clinical model (n = 83; events = 53; AIC = 394)^*^ | | Clinical model + pre-therapeutic MRI (n = 82; events =52; AIC = 383) | | Clinical model + pre-therapeutic MRI + post-therapeutic MRI (n = 81; events = 51; AIC = 373) | |
| Therapy group: TKI | 0.283 | 0.739 (0.425-1.284) | 0.875 | 0.953 (0.526-1.729) | 0.724 | 0.118 (0.603-2.072) |
| Liver metastases | 0.059 | 1.920 (0.974-3.787) | 0.080 | 1.848 (0.929-3.677) | 0.130 | 1.742 (0.850-3.569) |
| Cyfra 21.1 | 0.051 | 1.024 (0.999-1.048) | 0.134 | 1.019 (0.994-1.044) | 0.096 | 1.022 (0.996-1.049) |
| ECOG = 0 | 0.025 | 0.532 (0.306-0.925) | 0.048 | 0.568 (0.324-0.985) | 0.050 | 0.568 (0.322-1.000) |
| Tumor pre-therapeutic MRI  Slope 0s-70s [*10] |  |  | 0.025 | 0.889 (0.801-0.985) | 0.090 | 0.911 (0.817-1.015) |
| Tumor post-therapeutic MRI  Slope 0s-40s [*10] |  |  |  |  | 0.117 | 0.940 (0.870-1.016) |

Supplementary Table A.3. Multivariate Cox Regression of FAMoS selected variables referring to PFS

|  | P-value | Hazard Ratio (95% CI) | P-value | HR | P-value | Hazard Ratio (95% CI) |
| --- | --- | --- | --- | --- | --- | --- |
|  | Clinical model (n = 83; events = 76; AIC = 529) | | Clinical model + pre-therapeutic MRI | | Clinical model + pre-therapeutic MRI + post-therapeutic MRI (n = 96, events = 85; AIC = 618) | |
| Therapy group: TKI | 0.003 | 0.477 (0.295-0.773) |  |  | 0.028 | 0.608 (0.390-0.946) |
| Liver metastases | 0.028 | 2.055 (1.082-3.904) |  |  | <0.001 | 3.079 (1.757-5.396) |
| Cyfra 21.1 | 0.077 | 1.018 (0.998-1.038) |  |  | Cyfra 21.1 no benefit | |
| ECOG = 0 | 0.035 | 0.595 (0.368-0.964) |  |  | 0.012 | 0.568 (0.322-1.000) |
|  |  |  | Pre-therapeutic MRI no benefit | | Pre-therapeutic MRI no benefit | |
| Tumor post-therapeutic MRI  Rel. Uptake 40s |  |  |  |  | <0.001 | 0.976 (0.963-0.990) |
